# Thromboelastography-Guided Goal-Directed Transfusion During Hepatectomy in a Fontan Patient With Life-Threatening Hemorrhage: A Case Report

**DOI:** 10.7759/cureus.107173

**Published:** 2026-04-16

**Authors:** Tokimitsu Hibino, Hitomi Tokuyama, Yusuke Okui, Shinichiro Irabu, Yoshie Toba

**Affiliations:** 1 Department of Public Health, International University of Health and Welfare, Tokyo, JPN; 2 Department of Anaesthesiology, Seirei Hamamatsu General Hospital, Hamamatsu, JPN; 3 Department of Emergency and Critical Care Medicine, Seirei Hamamatsu General Hospital, Hamamatsu, JPN; 4 Department of Hepatobiliary and Pancreatic Surgery/Acute Care Surgery, Seirei Hamamatsu General Hospital, Hamamatsu, JPN

**Keywords:** fontan-associated liver disease, fontan circulation, left hepatectomy, massive intraoperative hemorrhage, thromboelastography (teg)

## Abstract

Fontan-associated liver disease (FALD) is a critical long-term complication following Fontan surgery for complex congenital heart disease. The non-physiological Fontan circulation, which relies on elevated central venous pressure (CVP) to drive pulmonary blood flow, inherently leads to chronic hepatic congestion, fibrosis, and potentially hepatocellular carcinoma. We report the anesthetic management of a 25-year-old male with FALD and hepatocellular carcinoma (8.3 cm) undergoing open hepatectomy. Our anesthetic strategy focused on maintaining cardiac contractility, preserving venous return, and reducing pulmonary vascular resistance using inotropic and inodilator support and inhaled nitric oxide. During liver resection, the patient experienced catastrophic hemorrhage (6,875 mL) characterized by diffuse oozing due to high CVP and coagulopathy, leading to hypovolemic shock. Hemodynamics were sustained by ultra-rapid transfusion (up to 200 mL/min) and vasopressor support. Intraoperative coagulation was monitored via thromboelastography (TEG), which revealed severe qualitative and quantitative deficiencies in fibrinogen function that persisted despite fresh frozen plasma transfusion. Targeted administration of 3 g of fibrinogen concentrate and 40 units of platelet concentrate based on TEG findings achieved clinical hemostasis. Despite the massive bleeding and postoperative heart failure, the patient was successfully discharged. In conclusion, this case suggests that for hepatectomy in Fontan patients, a multidisciplinary approach incorporating the Pringle maneuver and TEG-guided precision transfusion can be a vital strategy to prevent circulatory collapse and manage life-threatening hemorrhage.

## Introduction

Fontan-associated liver disease (FALD) is a critical long-term complication following the Fontan procedure [1]. This surgery serves as a functional repair for complex congenital heart defects where biventricular repair is precluded. By directing systemic venous return directly to the pulmonary arteries, the procedure effectively mitigates cyanosis [2]. While this innovation has dramatically improved survival, with 10-year survival rates reaching 98% since 2001 [3], the resulting Fontan circulation is inherently non-physiological.

Lacking a subpulmonary pump, the circulation relies entirely on the pressure gradient between an elevated central venous pressure (CVP) and a low left atrial pressure. This chronically high CVP imposes a significant pressure load on the hepatic veins and the central veins of the hepatic lobules. Histologically, this leads to sinusoidal dilatation, inflammatory infiltration, perivenular fibrosis, and hepatocyte atrophy, ultimately progressing to cirrhosis [1].

FALD is nearly universal over time; a systematic review by Cao et al. suggests its presence in all patients five to 10 years following the Fontan procedure [4]. The risk is progressive, with the incidence of hepatocellular carcinoma (HCC) reaching 4.9% within 20 years [5]. Notably, Inuzuka et al. demonstrated that every 3 mmHg increase in CVP correlates with a 31% increase in the risk of cirrhosis and HCC [6].

Performing liver resection in Fontan patients presents formidable challenges. The physiological hallmark of the Fontan circulation, a persistently elevated CVP, creates a high-pressure gradient across the hepatic venous system. Unlike the typical low-pressure environment of the hepatic veins in biventricular circulation, the Fontan liver is under constant "back-pressure." During hepatic transection, this elevated venous pressure precludes the use of standard low-CVP techniques often employed in liver surgery to minimize blood loss. Consequently, even minor vascular injuries can lead to rapid, high-volume retrograde hemorrhage from the hepatic veins, which lack functional valves to impede this flow. High venous pressure increases the risk of catastrophic bleeding, which is further complicated by unique coagulopathies and limited cardiac reserve [7]. Furthermore, traditional coagulation tests (e.g., fibrinogen levels, platelet counts) often fail to capture the complex, "rebalanced" yet fragile hemostatic state of Fontan patients. While massive hemorrhage protocols typically rely on fixed-ratio transfusions, such empirical strategies may be inadequate or even hazardous in this population, potentially leading to volume overload and Fontan circuit failure. There is a critical need for real-time, functional assessment of clot formation to guide precise intervention. In this report, we highlight the unique utility of thromboelastography (TEG)-guided transfusion as a valuable tool for managing catastrophic bleeding in a Fontan patient - a clinical application that has been sparsely documented in this specific high-risk surgical context.

## Case presentation

Preoperative status and preparation

**Figure 1:**
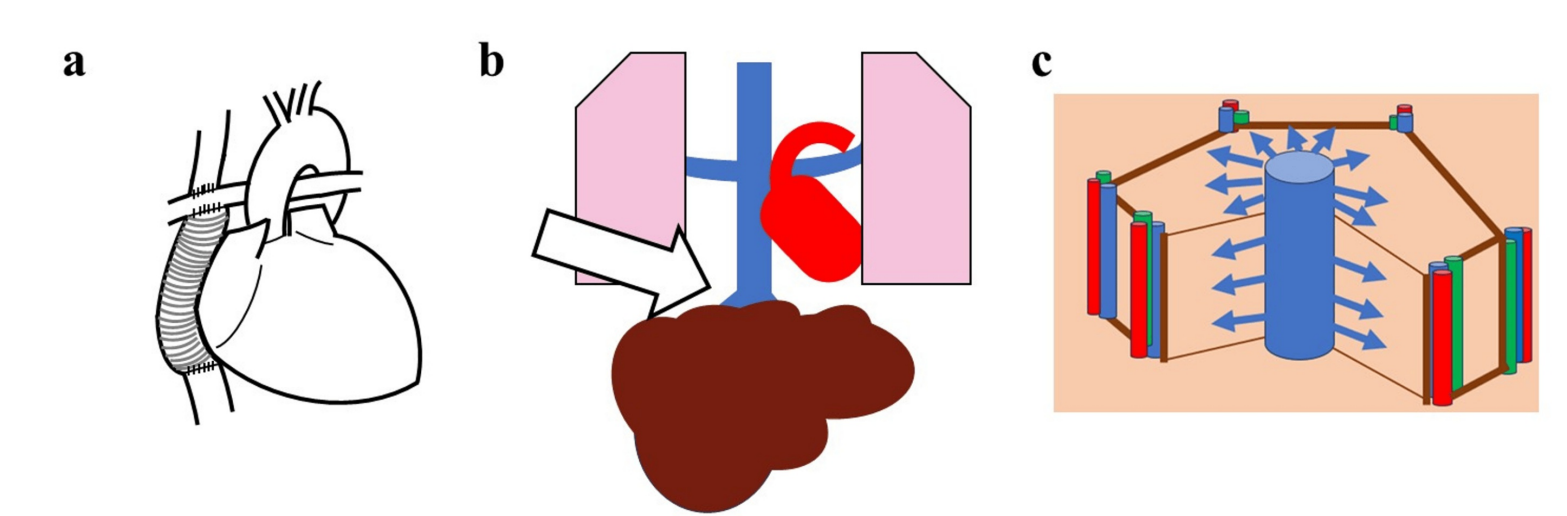
Cardiac anatomy and the pathophysiology of Fontan-associated liver disease (FALD) (a) Anatomical illustration of the Fontan circulation (total cavopulmonary connection with an extracardiac conduit). (b) Schematic of the pathophysiology in Fontan circulation, where elevated central venous pressure is directly transmitted to the liver (white arrow) due to the absence of a subpulmonary ventricle. (c) Schematic representation of a liver lobule. The portal triads, consisting of the hepatic artery (red), portal vein (blue), and bile duct (green), are located at the corners of the hexagonal lobule. Blue arrows indicate the direction of high central venous pressure transmitted from the central vein to the sinusoids. Image credits: Created by the authors using Microsoft PowerPoint (version 2508, build 16.0.19127.20302; Microsoft Corp., Redmond, WA, USA).

A 25-year-old male of mixed Japanese and South American descent (weight 52.5 kg, BSA 1.52 m^2^) with the Fontan circulation was referred to our hospital in 2020 following the discovery of a left hepatic lobe tumor. His complex cardiac history included a multiperforated muscular ventricular septal defect (VSD), double-orifice mitral valve, hypoplastic aortic arch, and aortic coarctation. Due to the infeasibility of biventricular repair, he underwent six palliative surgeries, starting with pulmonary artery banding and aortic arch reconstruction at three months of age. His surgical course included a lateral tunnel total cavopulmonary connection (TCPC) at age 2, followed by a conversion to an extracardiac conduit at age 3 due to a baffle leak (Figure 1). Subsequent interventions addressed VSD enlargement, tricuspid regurgitation, and recurrent arrhythmias, eventually requiring epicardial pacemaker implantation at age 22.

**Figure 2:**
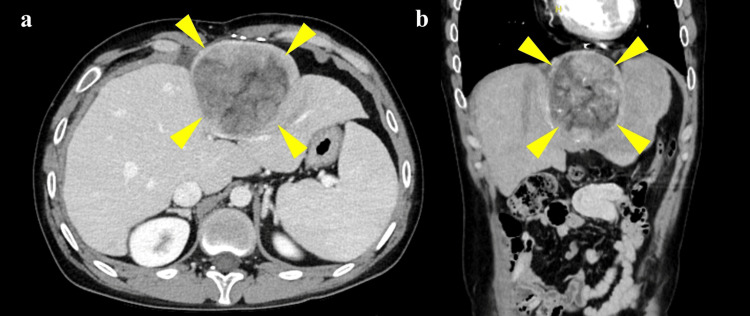
Contrast-enhanced abdominal computed tomography (CT) revealing a massive liver tumor (a) Axial and (b) coronal contrast-enhanced CT images showing a massive liver tumor (approximately 8.3 cm in diameter; yellow arrowheads) occupying the left lobe of the liver.

The liver tumor, located in segment S2, measured 7.4 × 7.4 × 8.3 cm (Figure 2). Open hepatectomy was chosen over a laparoscopic approach due to the tumor’s size. Preoperatively, the patient’s liver function was Child-Pugh class A (six points), with a Model for End-Stage Liver Disease excluding INR (MELD-XI) of 10.0. Although epidural anesthesia was planned to facilitate early extubation and maintain spontaneous breathing - crucial for Fontan hemodynamics - the baseline platelet count was 81 × 10^9^/L. To mitigate the risk of epidural hematoma, 20 units of platelet concentrate (PC) were transfused prior to epidural anesthesia (Table 1). We recognize that, considering the risk of volume overload in Fontan hemodynamics, fibrinogen concentrate guided by TEG would have been an ideal, volume-sparing alternative. However, the patient’s preoperative fibrinogen level (215 mg/dL) did not meet the national insurance criteria for fibrinogen concentrate (typically <150 mg/dL), and TEG assessment was restricted to intraoperative use by institutional policy. Thus, we prioritized conventional platelet transfusion to safely perform epidural catheterization. Epidural anesthesia was performed at the T9-T10 interspace with the patient in the lateral decubitus position. A catheter was advanced 5 cm cranially, and an initial dose of 4 mL of 0.375% ropivacaine with 2 mg of morphine was administered. To maintain analgesia while minimizing the risk of a high thoracic block, which could impair cardiac sympathetics (T1-T4) and diaphragmatic function (C3-C5), a continuous infusion of 0.25% ropivacaine was initiated at 4 mL/h. However, following the onset of subsequent catastrophic hemorrhage, the epidural infusion was immediately discontinued to prioritize systemic vascular resistance and hemodynamic stability.

**Table 1:** Preoperative laboratory data Bold values with arrows (↑/↓) indicate results outside the reference range. Reference ranges are based on the standards of our institution (Seirei Hamamatsu General Hospital). AFP: alpha-fetoprotein; ALP: alkaline phosphatase; ALT: alanine aminotransferase; aPTT: activated partial thromboplastin time; AST: aspartate aminotransferase; CRP: C-reactive protein; eGFR: estimated glomerular filtration rate; LDH: lactate dehydrogenase; PT-INR: prothrombin time-international normalized ratio

Blood Test Items	Values	Reference Range
Sodium	137 mmol/L (↓)	138-145 mmol/L
Potassium	3.9 mmol/L	3.6-4.8 mmol/L
Chloride	104 mmol/L	101-108 mmol/L
Total protein	75 g/L	66-81 g/L
Albumin	51 g/L	41-51 g/L
Total bilirubin	34.2 μmol/L (↑)	6.8-25.7 μmol/L
AST	37 U/L (↑)	13-30 U/L
ALT	41 U/L	10-42 U/L
LDH	185 U/L	124-222 U/L
ALP	112 U/L	38-113 U/L
CRP	0.04 mg/dL	0.00-0.14 mg/dL
AFP	96.76 ng/mL (↑)	0-8.78 ng/mL
Creatine kinase	52 U/L (↓)	59-248 U/L
Blood urea nitrogen	5.7 mmol/L	2.9-7.1 mmol/L
Creatinine	58.7 μmol/L	49.6-81.6 μmol/L
eGFR	103 mL/min/1.73m^2^	>60 mL/min/1.73m^2^
White blood cell count	5.5 x 10^3^/μL	3.3-8.6 x 10^3^/μL
Hemoglobin	167 g/L	137-168 g/L
Hematocrit	50.10%	40.7-50.1%
Platelet count	81 x 10^9^/L (↓)	158-348 x 10^9^/L
PT-INR	1.02	0.85-1.10
aPTT	28.8 s	24-34 s
Fibrinogen	215 mg/dL	200-400 mg/dL

Anesthesia induction and early intraoperative events

In the operating room, standard monitoring was supplemented with a central venous catheter (CVC) in the right internal jugular vein and transesophageal echocardiography. While objective EEG-based monitoring (e.g., bispectral index or entropy) was intended to be used to ensure adequate hypnotic depth, it was unavailable due to an unexpected equipment failure. Consequently, anesthetic depth was clinically monitored through hemodynamic trends and autonomic responses. Anesthesia was induced with ketamine (50 mg), propofol (60 mg), rocuronium (50 mg), and a remifentanil infusion at 0.4 μg/kg/min. Maintenance was achieved with 2-3% desflurane and remifentanil at the same rate, supplemented by a continuous rocuronium infusion at 0.29 mg/kg/h.

We targeted a train-of-four (TOF) count of 1-2 to avoid progressing to a deep neuromuscular blockade caused by potential rocuronium accumulation. However, the patient exhibited sudden "bucking" 30 minutes after induction as the TOF count increased. This episode underscored that clinical parameters alone were insufficient to reliably predict motor responses to intense surgical stimuli, particularly when objective sedation monitoring was unavailable. To ensure surgical safety and immobility, an additional 30 mg of rocuronium was administered, and the infusion rate was increased to 0.39 mg/kg/h, maintaining a TOF count of 0 thereafter.

The remifentanil dose was set at 0.4 μg/kg/min to ensure a prompt and smooth induction. Given the patient’s young age, we opted for this dose to avoid a prolonged induction period. A slow, incremental titration of remifentanil could have delayed the loss of consciousness and paradoxically increased the risk of opioid-induced muscle rigidity (the "lead-pipe" phenomenon), potentially complicating airway management. Therefore, a decisive initial dose was prioritized to achieve adequate anesthetic depth rapidly while balancing the patient’s cardiovascular stability. Inotropic and inodilator support, including dopamine (2 μg/kg/min), dobutamine (2 μg/kg/min), and milrinone (0.5 μg/kg/min), was initiated via the CVC to maintain cardiac output and stabilize hemodynamics against anesthesia-induced depression and surgical stress. Milrinone was utilized for its dual effects of enhancing ventricular lusitropy and reducing pulmonary vascular resistance, both of which are critical for maintaining passive pulmonary blood flow in the Fontan circulation.

**Figure 3:**
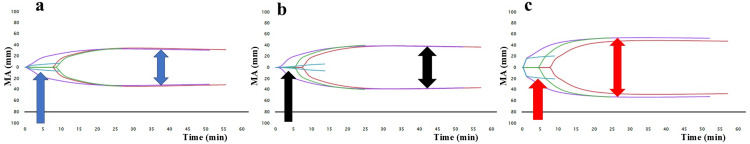
Serial thromboelastography (TEG) traces TEG evaluates the viscoelastic properties of whole blood during clot formation. The time from the start of the test until the initial fibrin formation (clotting time) reflects coagulation factor activity, while the MA represents the ultimate strength of the fibrin-platelet clot. In this case, we focused on two key parameters: citrated functional fibrinogen MA (CFF.MA; blue tracing), which isolates fibrinogen function, and citrated rapid TEG MA (CRT.MA; purple tracing), which reflects the combined contribution of platelets and fibrinogen to clot strength. The presented waveforms represent the final results displayed by the TEG 6s system upon completion of all measurement channels. (a) Preoperative baseline. The CFF.MA is low, suggesting impaired fibrinogen function (blue arrow). The CRT.MA is also small, suggesting reduced clot strength (double blue arrows). (b) During massive hemorrhage, demonstrating a reduction in CFF.MA (black arrow). The CRT.MA remains low, suggesting that clot strength has not recovered (double black arrows). (c) After administration of 3 g fibrinogen concentrate and 40 units of platelets, showing remarkable improvement. Both CFF and CRT MAs have returned to the normal range, indicating adequate fibrinogen function (red arrow) and restored clot strength (double red arrows). The contrast with panels (a) and (b) clearly illustrates the severe preoperative and intraoperative deficits. CFF: citrated functional fibrinogen; CRT: citrated rapid TEG; MA: maximum amplitude

The initial TEG (TEG® 6s Hemostasis System, Haemonetics Corp., Boston, MA, USA) showed normal coagulation factor activity (CK.R 8.1 minutes; reference range: 4.6-9.1 minutes) but remarkably impaired platelet (CRT.MA 32.1 mm; reference range: 52-78 mm) and fibrinogen function (CFF.MA 7.0 mm; reference range: 15-32 mm), based on the manufacturer’s reference ranges (Figure 3a). Forty minutes into the surgery, shortly after the initiation of inotropic support and an episode of patient bucking, the patient developed atrial fibrillation. This arrhythmia was likely triggered by the sympathomimetic effects of dobutamine and dopamine, potentially exacerbated by the acute sympathetic surge associated with the bucking episode under a Fontan-related fragile electrical substrate. This event resulted in hemodynamic instability, with blood pressure dropping to 90/45 mmHg and heart rate reaching 110 bpm.

Hepatic resection and catastrophic hemorrhage

**Figure 4:**
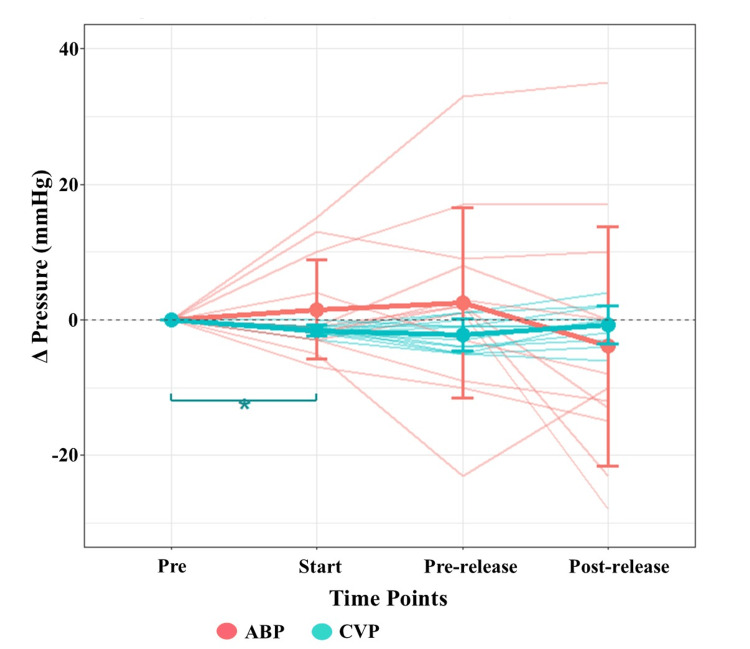
Changes in arterial blood pressure (ABP) and central venous pressure (CVP) during the Pringle maneuver The graph illustrates the mean changes (Δ) in ABP (red) and CVP (blue) at four time points: pre-Pringle (Pre), start of the maneuver (Start), immediately before release (Pre-release), and after release (Post-release). Data are presented as mean ± standard deviation. Statistical significance was determined using the Wilcoxon signed-rank test with Bonferroni correction for multiple comparisons between time points. The asterisk (*) indicates a statistically significant difference (p < 0.05). The statistical analyses were performed using EZR version 1.68 (Easy R, Saitama Medical Center, Jichi Medical University, Saitama, Japan), which is a graphical user interface for R version 4.3.1 (The R Foundation for Statistical Computing, Vienna, Austria) [8]. Data are presented as descriptive statistics to illustrate the hemodynamic fluctuations associated with repeated Pringle maneuvers. No inferential claims were made regarding the general population. The p-values calculated in this report are not intended for generalization to other patients. Instead, they were used to verify that the observed differences in physiological parameters during and between repeated Pringle maneuvers within this specific case were not due to random chance, but reflected consistent physiological responses.

**Figure 5:**
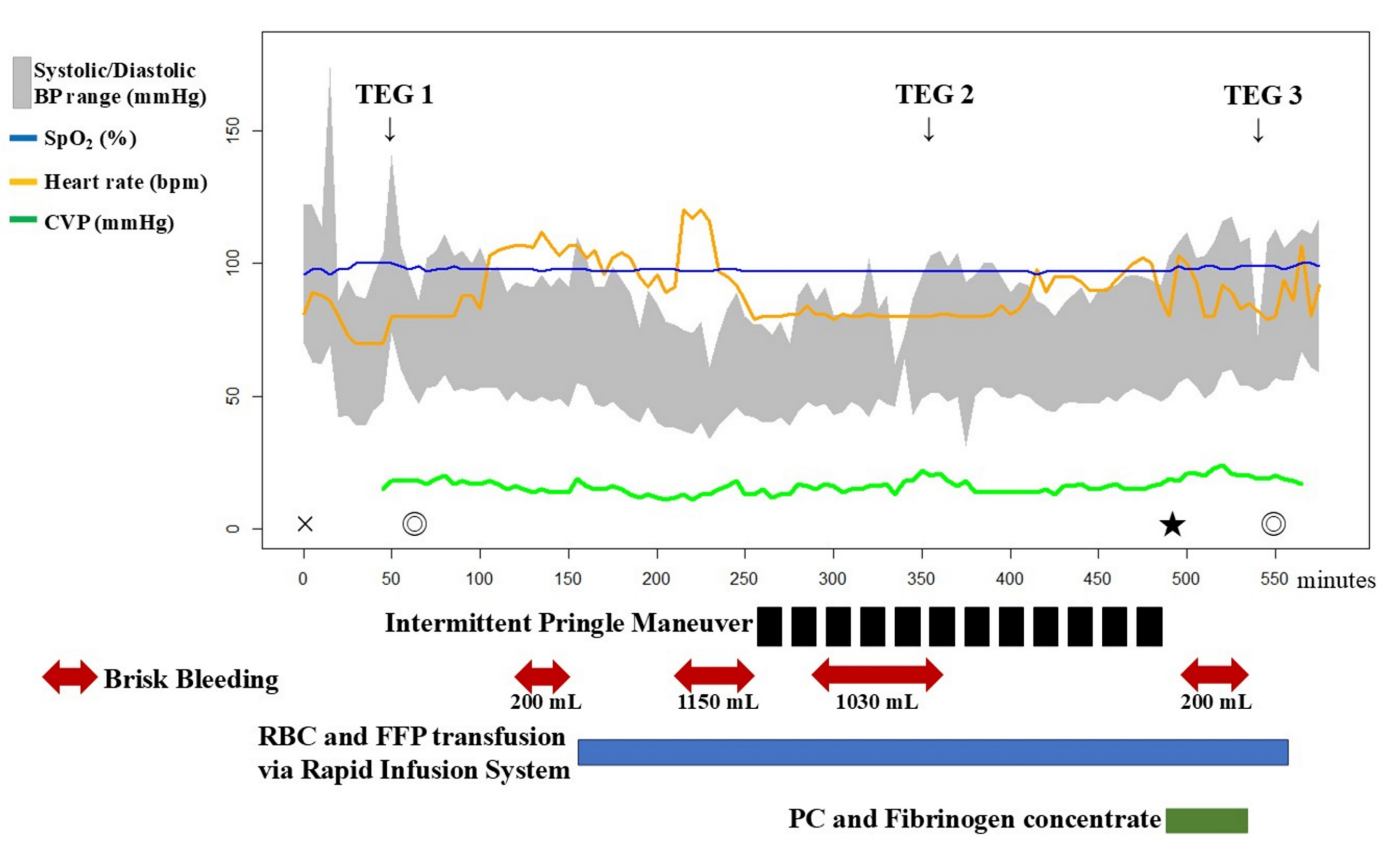
Intraoperative anesthetic record The gray-shaded area represents the systolic and diastolic BP range, while the blue, orange, and green lines indicate SpO_2_, heart rate, and CVP, respectively. Thromboelastography (TEG 6s; Haemonetics, Braintree, MA, USA) was used for real-time coagulation monitoring (arrows with TEG 1-3). TEG 1 was obtained after anesthesia induction as a baseline control. TEG 2 was performed when cumulative blood loss reached 2,500 mL to comprehensively evaluate the progressing coagulopathy. TEG 3 was conducted following the completion of fibrinogen concentrate and platelet transfusion to assess the necessity for further hemostatic intervention. The cross (×) indicates the start of anesthesia induction, and the double circles (◎) indicate the start and end of surgery. The star (★) denotes the timing of specimen retrieval. Intermittent Pringle maneuvers (black blocks) were performed during liver resection. Brisk bleeding (red double-headed arrows with loss volumes) was managed with warmed RBC and FFP transfusion via a rapid infusion system (Belmont Rapid Infuser RI-2; Belmont Instrument Corp., Billerica, MA, USA) (blue bar). BP: blood pressure; CVP: central venous pressure; FFP: fresh frozen plasma; PC: platelet concentrate; RBC: red blood cells; SpO_2_: oxygen saturation

During liver dissection, bleeding became catastrophic despite the Pringle maneuver (performed 12 times; see Figure 4 for CVP/blood pressure dynamics). Data visualization and clinical parameter trends were analyzed using EZR (Easy R, Saitama Medical Center, Jichi Medical University, Saitama, Japan) (Figure 4) [8]. Within the first 30 minutes of dissection, blood loss reached 1150 mL. A rapid infusion system (Belmont® Rapid Infuser RI-2, Belmont Medical Technologies, Billerica, MA, USA) was used to deliver red blood cells (RBCs) and fresh frozen plasma (FFP) at rates up to 200 mL/min (Figure 5). To counteract citrate-induced hypocalcemia during this ultra-rapid transfusion, 60 mL of 2% calcium chloride was administered in divided 10-mL boluses. Ionized calcium levels were closely monitored and maintained between 0.80 and 1.13 mmol/L, ensuring the preservation of both myocardial contractility and the coagulation cascade. To maintain systemic blood pressure and counteract the vasodilatory effects of milrinone and dobutamine during this catastrophic hemorrhage, vasopressor support was escalated with norepinephrine (up to 0.24 μg/kg/min) and vasopressin (3 U/h). These agents were titrated to support systemic vascular resistance and ensure adequate perfusion pressure despite the massive volume loss. As CVP rose above 20 mmHg due to massive transfusion, inhaled nitric oxide (iNO) (20 ppm) was started to reduce pulmonary vascular resistance and support the Fontan circuit. A second TEG at a cumulative blood loss of 2500 mL revealed further deterioration of fibrinogen function (CFF.MA 6.2 mm) despite prior 10 units of FFP administration (Figure 3b). To address the severe deficits in fibrinogen and platelet function, 3 g of fibrinogen concentrate and 40 units of PC were administered following the completion of liver tumor resection. A third TEG confirmed the normalization of all parameters (CK.R 8.0 s, CRT.MA 52.4 mm, CFF.MA 20.6 mm), and clinical hemostasis was achieved (Figure 3c).

Total blood loss was 6875 mL, with a total transfusion volume of 7240 mL (RBC 22 units, FFP 20 units, PC 40 units, fibrinogen concentrate 3 g). Regarding oxygenation, the preoperative PaO_2_/FiO_2_ (P/F) ratio was 338. Intraoperative arterial blood gas analysis was performed at key intervals: at the start of liver resection, 10 minutes after initiating iNO, and at the conclusion of the surgery. The P/F ratios were 298.3, 290.0, and 298.6, respectively, showing a slight decrease compared to preoperative values. Postoperative chest radiography revealed prominent pulmonary artery shadows and congestion; however, the lung fields remained clear without evidence of infiltrates or decreased translucency, making pulmonary edema or transfusion-related acute lung injury (TRALI) unlikely. Nevertheless, since these findings were obtained under mechanical ventilation with a positive end-expiratory pressure of 5 cmH_2_O, the risk of developing acute pulmonary edema following extubation could not be entirely excluded. Consequently, extubation was deferred until postoperative day 1 (POD1) to ensure respiratory stability. He was successfully discharged from the intensive care unit on POD 2.

Postoperative course and follow-up

After adjusting heart failure medications and pacemaker settings, he was discharged on POD 52 without sequelae. Nine months postoperatively, sternal metastasis was identified, and radiation therapy was initiated. Eleven months postoperatively, the patient was emergently transported to a local general hospital due to a sudden headache and impaired consciousness. He was diagnosed with cerebellar hemorrhage from a brain metastasis and underwent emergency hematoma evacuation. After his condition stabilized, he was transferred back to our hospital. Although further radiotherapy and chemotherapy were considered, the patient's deteriorating systemic status led to the decision for best supportive care. As of 14 months postoperatively, the patient remains alive, though his prognosis is extremely poor.

## Discussion

Management of massive hemorrhage

Liver resection in a patient with the Fontan circulation presents a unique hemodynamic challenge. The chronic elevation of CVP causes not only hepatic congestion but also diffuse venous oozing from the surgical field, complicating surgical hemostasis. In this case, we encountered catastrophic bleeding (6875 mL), exceeding previously reported ranges of 1000-4130 mL for similar procedures (Table 2) [9-15]. Our management strategy focused on minimizing pulmonary vascular resistance through milrinone and iNO to facilitate Fontan flow. However, the sheer rate of blood loss necessitated continuous, ultra-rapid transfusion at speeds up to 200 mL/min. Despite transient hypotension (60/30 mmHg), irreversible circulatory collapse was averted by (1) the patient’s preserved left ventricular morphology, which offers superior stress tolerance compared to right ventricular dominance [16]; (2) optimized cardiac contractility via multimodal inotropic support; and (3) a multidisciplinary clinical team capable of managing prolonged rapid transfusion.

**Table 2:** Reported cases of open liver resection in Fontan patients AFP: alpha-fetoprotein; ALP: alkaline phosphatase; ALT: alanine aminotransferase; AST: aspartate aminotransferase; ECMO: extracorporeal membrane oxygenation; PT-INR: prothrombin time-international normalized ratio; NR: not reported; TEG: thromboelastography

Case Characteristics	Weyker et al. (2014) [9]	Kwon et al. (2015) [10]	Takuma et al. (2016) [11]	Lo et al. (2018) [12]	Nemoto et al. (2020) [13]	Sumie et al. (Case 1) (2022) [14]	Sumie et al. (Case 2) (2022) [14]	Sumie et al. (Case 3) (2022) [14]	Ho et al. (2024) [15]	Our Case (2026)
Age	23	32	29	24	37	21	28	43	24	25
Sex	Female	Male	Female	Female	Female	Female	Female	Male	Female	Male
Total bilirubin (μmol/L)	NR	19	17	NR	NR	NR	NR	NR	22	34.2
Albumin (g/L)	NR	48	49	NR	NR	NR	NR	NR	44	51
Creatinine (μmol/L)	NR	69.4	60.2	NR	NR	NR	NR	NR	62	58.7
PT-INR	NR	2.2	1.68	1.3	NR	1.28	1.03	1.23	1.3	1.02
Platelet count (10^9^/L)	NR	225	125	104	NR	184	174	139	104	81
AST (U/L)	NR	23	23	NR	NR	NR	NR	NR	38	37
ALT (U/L)	NR	21	13	NR	NR	NR	NR	NR	22	41
AFP (ng/mL)	NR	13000	117.1	>5000000	81663	NR	NR	NR	33897	96.76
Child-Pugh	NR	A	A	A	A	A	A	A	A	A
Fenestration	NR	Absent	NR	Absent	NR	Absent	NR	Absent	Present	Absent
Perioperative CVP (mmHg)	NR	10-15	NR	14	12	10-15	15-20	13-20	NR	11-24
Tumor size (cm)	14.8	4.2	1.5	6.8	6.3	3.6	8	2.3	9.5	8.3
Blood loss (mL)	1000	1500	NR	4100	3200	2650	1181	2850	4130	6875
Pringle maneuver	Not performed	Performed	NR	Performed	Performed	NR	NR	NR	Not performed	Performed
Postoperative hospital stay (d)	NR	9	NR	12	9	NR	NR	NR	12	52
Key features	Venovenous bypass used	Preserve 900 mL of autologous blood	Unremarkable	ECMO standby (Not used)	Reverse Trendelenburg position	Rectus sheath block	Transversus abdominis plane block	Conversion from laparoscopic to open surgery	Cell salvage used	TEG used

The role of the Pringle maneuver

In this case, the Pringle maneuver was employed to occlude hepatic artery and portal vein blood flow, aiming to control bleeding from the hepatic resection margin. Theoretically, the Pringle maneuver in a Fontan patient carries a dual risk: it could lead to systemic hypoperfusion by reducing venous return (preload), while simultaneously failing to control bleeding if the high CVP causes significant retrograde flow from the hepatic veins.

However, our clinical observations provided valuable insights into these dynamics. As shown in Figure 4, while CVP decreased significantly immediately after initiating the Pringle maneuver, systemic blood pressure remained remarkably stable both during the occlusion and after its release. Furthermore, the significant CVP reduction observed during the Pringle maneuver suggests its direct contribution to lowering the "back-pressure" at the hepatic resection surface. In patients with the Fontan circulation, CVP is chronically elevated (reaching 15-23 mmHg in this case), and this pressure is transmitted retrograde through the hepatic veins, which lack valves. This creates a state of persistent sinusoidal congestion. Even when the inflow (portal vein and hepatic artery) is occluded by the Pringle maneuver, the resection surface remains vulnerable to "back-flow bleeding" from the hepatic veins.

Choukèr et al. demonstrated that the Pringle maneuver can reduce CVP while maintaining mean arterial pressure [17]. Our findings align with this, suggesting that the maneuver mitigated the high-pressure gradient at the site of dissection. Given that Wang et al. emphasized the necessity of keeping CVP below 4 mmHg to minimize blood loss during hepatectomy [18], the management of Fontan patients, who inherently cannot reach such low CVP levels, requires every possible measure to bridge this gap. Therefore, we conclude that, in this specific case, the Pringle maneuver was not only safe from a hemodynamic standpoint but was also an indispensable component of our hemorrhage control strategy. Without it, the total blood loss would likely have been even more catastrophic.

Coagulation management using TEG

While physical blood flow control via the Pringle maneuver was effective, the catastrophic hemorrhage, requiring an ultra-rapid infusion rate of 200 mL/min, necessitated immediate countermeasures against the concurrently progressing consumptive coagulopathy. In this critical situation, real-time monitoring with the TEG® 6s Hemostasis System facilitated our management.

Fontan patients are known to have a 19% to 35% reduction in most coagulation factors compared to healthy individuals, predisposing them to bleeding disorders [19]. Paradoxically, reduced protein S levels, impaired fibrinolysis, endothelial dysfunction, and chronic platelet activation create a prothrombotic state. This results in a precarious coagulation-fibrinolysis balance [19]. Consequently, massive hemorrhage can rapidly deplete these already limited factors, leading to severe coagulopathy. Conversely, attempting to promote hemostasis with agents such as recombinant activated factor VII may carry a substantial risk of devastating thrombosis. Furthermore, as we have previously reported, plasma fibrinogen levels in Fontan patients do not always correlate with the functional fibrinogen contribution to clot strength (CFF.MA), often masking severely impaired fibrinogen function [20].

In the present case, three factors complicated coagulation management: (1) the inherently unstable coagulation profile associated with Fontan physiology; (2) the discrepancy between conventional laboratory parameters, such as fibrinogen levels and platelet counts, and actual functional clot strength; and (3) the exacerbation of these imbalances by massive intraoperative hemorrhage and subsequent hemodilution. We faced a constant clinical dilemma between prioritizing volume replacement for circulatory stability and minimizing CVP to prevent venous congestion. The necessary volume resuscitation during massive bleeding further induced hemodilution, making it increasingly difficult to assess the patient's true coagulation status through static laboratory data alone. Consequently, it was clinically rational to evaluate not only the plasma levels of clotting factors but also the actual clot initiation time and strength, necessitating TEG-guided therapy for precise and timely intervention.

The preoperative baseline TEG revealed a CFF.MA of 7.0 mm. While the serum fibrinogen level was mildly below the normal range at 167 mg/dL, it did not fully account for the severe impairment in clot strength. This discrepancy between the quantitative measurement and functional capacity suggests a qualitative fibrinogen abnormality (Figure 3a). During the surgery, a second TEG was performed after the initial massive bleeding had been stabilized by the administration of 10 units each of RBCs and FFP. Despite the FFP transfusion, the CFF.MA further deteriorated to 6.2 mm (fibrinogen 135 mg/dL) (Figure 3b), confirming that FFP alone was insufficient to restore fibrinogen function. This result serves as a clear clinical example of "FFP futility" in correcting specific fibrinogen deficits during massive hemorrhage, where the volume-heavy replacement likely exacerbated hemodilution. Therefore, earlier use of fibrinogen concentrate should be considered as a more volume-sparing strategy, provided that hemodynamic stability is maintained and regulatory or institutional constraints regarding its administration are met. Accordingly, we administered 3 g of fibrinogen concentrate and 40 units of PC.

Following this targeted replacement, clinical hemostasis in the surgical field markedly improved. A third TEG was then performed to ensure that the patient had not transitioned into a hypercoagulable state. This showed a CFF.MA of 20.6 mm (fibrinogen 274 mg/dL), confirming that fibrinogen and platelet functions had been restored to within normal ranges without over-correction (Figure 3c). This experience underscores that, at least in this case, TEG-based point-of-care monitoring was indispensable for accurately assessing coagulation status and executing a precise transfusion strategy for a Fontan patient facing life-threatening hemorrhage.

## Conclusions

This case suggests that successful hepatectomy is achievable within the complex physiological framework of the Fontan circulation, even in the face of catastrophic hemorrhage. Although the elevated CVP characteristic of this population poses a substantial risk of exacerbating intraoperative bleeding, physical inflow occlusion via the Pringle maneuver served as an effective strategy to mitigate venous pressure at the resection plane while maintaining hemodynamic stability. However, such management requires a delicate balance; while inotropic support may be necessary to maintain cardiac output, it must be cautiously titrated alongside vasopressors to minimize the risks of tachycardia and arrhythmias. Most notably, this case highlights the clinical utility of TEG for real-time coagulation management. The observation that CFF.MA continued to decrease despite the administration of 10 units of FFP, suggesting that conventional FFP-centric replacement strategies, guided by standard laboratory tests, may be inadequate during massive hemorrhage in Fontan patients with pre-existing coagulopathy. The targeted administration of fibrinogen concentrate was a rational intervention, rapidly restoring clot strength while avoiding unnecessary volume overload that could compromise the Fontan circuit.

We emphasize that in catastrophic scenarios, the immediate requirement for volume resuscitation to prevent circulatory collapse must be balanced against the risks of hemodilution. TEG-guided monitoring demonstrated its value for signaling the critical transition from emergency volume-based resuscitation to targeted hemostatic therapy. Notably, while the complete TEG profile can take over 60 minutes to fully mature, specific critical parameters, such as CFF.MA (10-15 minutes) and CRT.MA (approximately 30 minutes), are algorithmically finalized much earlier. This rapid availability of definitive functional data, even in the midst of hemorrhage, provides a crucial advantage for time-sensitive decision-making. Our experience suggests that TEG-guided coagulation management could be a key component of a successful hemostatic strategy for liver resection in patients with Fontan circulation.
